# Model-Based Analysis of Electrode Placement and Pulse Amplitude for Hippocampal Stimulation

**DOI:** 10.1109/TBME.2018.2791860

**Published:** 2018-01-25

**Authors:** Clayton S. Bingham, Gene J. Yu, Jean-Marie C. Bouteiller, Dong Song, Theodore W. Berger, Kyle Loizos, Andrew Gilbert, Gianluca Lazzi

**Affiliations:** University of Southern California, CA, USA; University of Southern California, CA, USA; University of Southern California, CA, USA; University of Southern California, CA, USA; University of Southern California, CA, USA; University of Utah, UT, USA; University of Utah, UT, USA; University of Utah, UT, USA

**Keywords:** Extracellular Electrical Stimulation, Admittance Method, Electrode Design, Multi-scale Modeling, NEURON

## Abstract

**Objective:**

The ideal form of a neural-interfacing device is highly dependent upon the anatomy of the region with which it is meant to interface. Multiple-electrode arrays provide a system which can be adapted to various neural geometries. Computational models of stimulating systems have proven useful for evaluating electrode placement and stimulation protocols, but have yet to be adequately adapted to the unique features of the hippocampus.

**Methods:**

As an approach to understanding potential memory restorative devices, an Admittance Method-NEURON model was constructed to predict the direct and synaptic response of a region of the rat dentate gyrus to electrical stimulation of the perforant path.

**Results:**

A validation of estimated local field potentials against experimental recordings is performed and results of a bi-linear electrode placement and stimulation amplitude parameter search are presented.

**Conclusion:**

The parametric analysis presented herein suggests that stimulating electrodes placed between the lateral and medial perforant path, near the crest of the dentate gyrus, yield a larger relative population response to given stimuli.

**Significance:**

Beyond deepening understanding of the hippocampal tissue system, establishment of this model provides a method to evaluate candidate stimulating devices and protocols.

## I. INTRODUCTION

In communicating with neural systems, there are few *in vivo* techniques more widely used than electrode-based stimulation and recording. One of the greatest feats of neural engineering has been the development of implantable medical devices which use multi-electrode arrays (MEAs) to record and stimulate neurons for the therapeutic treatment of brain disorders, whether through modulation (i.e. deep brain stimulation) or restoration (i.e. proposed hippocampal memory prosthesis) [1]. Advancements in medical implant technology have a trajectory toward “less and more”: smaller electronics and higher density MEAs [2,3,4]. However, the effectiveness of these engineering advances cannot be maximized without a fundamental understanding of the neural system that is the basis of the brain disorder, which includes the electrophysiology and anatomy of the neurons and their network topology. This knowledge can aid the design of MEAs by indicating the ideal location, orientation, number of electrodes, and stimulation pattern necessary to elicit a target response.

Damage to the hippocampus can result in memory impairment which profoundly affects the quality of life; to overcome these defects, a hippocampal memory prosthesis is being developed, and great strides have been made toward the prediction of hippocampal activity [1]. In this application, thorough quantitative evaluation of electrode arrays in terms of ability to efficiently elicit a targeted response is of particular importance due to the intricate, yet well documented, anatomy of the hippocampus. This intricacy may make it necessary to implement varied stimulating or recording electrode array geometries and complex pulsepatterns to elicit or detect a specific pattern of response from the excitable tissue. The ability to identify or generate a desired response can be dramatically enhanced by electrode arrays that are appropriately placed.

The difficulty of successfully identifying and quantifying neural activation by analyzing data from low-density LFP recordings, along with increased computational ability in recent years, has led to the construction of multi-scale computational models that include stimulating and/or recording electrodes. Virtual stimulation experiments of singular or simple network neuronal models have been used to study interactions between neural tissue and externally-applied electromagnetic fields. The modeling focus varies from prosthetic electrode design and effects of external stimulation [6–16], to assessing the accuracy of modern methodologies and parameters [17–21].

While these models have yielded promising electrode and modeling insights, more hippocampus specific biological realism is needed to satisfactorily evaluate electrode arrays for their ability to elicit particular spatiotemporal patterns of neural activation in the complex neural system. A virtual system is being developed with the goal of evaluating the efficacy of various electrode positions and stimulus patterns using a large-scale, three-dimensional Admittance MethodNEURON model of the dentate gyrus [6,7,22–25].

At a larger scale, the representation of extracellular space is vital to accurate prediction of the spatial distribution of current throughout the tissue and devices of a stimulating or recording system; therefore, accurate dielectric properties for discretizing the space into circuit components and distinct anatomical representations of the tissue are necessary [18,19].

Grill and McIntyre have made it clear that explicit neural models are advantageous because Rattay-style activating functions fail to accurately predict sites of action potential initiation [14,26]. While there are a range of possible individual neuronal models, with varying levels of detail being necessary to accurately recreate certain behaviors, it is unclear whether neural circuit responses to extracellular electrical stimulation can be accurately predicted with models much less complex than those described in this paper [27]. Due to preferential activation of neural subunits (dendrites, somas, and axons), where axonal chronaxie may be up to 40 times smaller than somatic, it seems important to implement complex and realistic morphologies which allow such spatial and anatomical specificity of activity [14,26,28]. Additionally, current source-sink dynamics between branching dendrite and cell body layers of laminated tissue create measurable dipoles in evoked potentials, features of which can be used for model validation against experimental recordings.

This work presents a systematic exploration of electrode location and stimulus amplitude in the dentate gyrus, which yields insights on the combined and individual influence of anatomy and stimulus amplitude on the population response. The availability of the state of every neuron, currently infeasible outside an *in silico* setting, allows the development of new metrics to describe the spatiotemporal network response and provides an initial surrogate investigation on describing activity *in vivo*, should the measurement of data at this scale become possible.

## II. METHODS

This paper presents the study of a model that combines both three-dimensional electric field calculation, via the Admittance Method (AM), and a NEURON model comprising 50,000 granule cells from Hendrickson et al., and 10,000 simplified perforant path axons [29]. These components are spatially arranged to approximate a three-dimensional region of the dentate gyrus. This combination Admittance Method-NEURON model is a powerful approach for computing neuronal response to extracellular fields because of its potential for modeling of not only the extracellular fields created by electrodes but also to provide a high-resolution view to the behavior of neural processes within that extracellular field.

### A. Defining the anatomical coordinate space

The hippocampal model presented herein was built following the segmented digital atlas of Kjonigsen et al. [30]. Their atlas contains high resolution images (1.4 pixels/μm) of 30 μm thick slices through the coronal plane of the rat brain, with each image digitally annotated to show the subdivisions of the hippocampus. While there are other established rodent brain atlases, such as those of Swanson or Paxinos, the present work is constructed using the Kjonigsen atlas because of the marginally higher resolution of their series of coronal slices, marked ease of use, and more complete region demarcation in the hippocampus [31,32]. A custom Matlab function was written to threshold each image, extracting the segmentation and converting the atlas to a voxelized format, where each voxel was assigned an index corresponding to its subdivision. A particularly clear slice image was taken at −3.34 mm from the bregma and the extracted boundaries were extruded 400 μm in the septotemporal direction, forming the entire 3dimensional space within which the AM-NEURON model was constructed.

### B. Admittance Method model construction

An in-house multi-resolution variant of the quasi-static AM was used to simulate the resulting voltage at nodes throughout this model due to given current input(s) [6,7,23–25].

In this method, a hexahedral, structured mesh is constructed in which the original voxel size is maintained near boundaries or predefined current source locations. As the distance from boundaries and sources is increased, voxels are merged. A maximum merged element size of 64 voxels was used. The original resolution of 12 μm-wide cubic voxels was implemented within the dentate gyrus, maintaining fine resolution in the tissue containing the superimposed NEURON model of the dentate gyrus. Once a mesh is constructed, it is overlaid with linear lumped circuit representations of resistors and capacitors based on the dielectric properties of the tissue and electrodes. Current sources are added into this network, which may be defined between any two nodes in the model. The resulting circuit was solved with the bi-conjugate gradient method with a residue of 10^−8^ as the stop criteria. Further details are provided in Cela [23].

The AM was chosen over alternative methods, such as the Finite Element Method (FEM), due to case-specific advantages exploited in this work. First, the method is relatively simple to implement relative to FEM. While FEM has the advantage of non-conformal grids, computational complexity grows with the number of independent current sources. Using a circuit model allows efficient inclusion of multiple current sources. This is a useful feature for modeling multi-electrode arrays and estimating feedback potentials. Second, the circuit description of the model space leads to an intuitive method for describing bulk neural tissue. This creates a more seamless integration with neural models, also represented by circuit components, by allowing for voltage at nodes within the circuital network of the volume conductor to be applied as extracellular voltage sources for neuronal models. This feature produces a coupled circuital description of bulk tissue and underlying neural networks. Further, arbitrary circuit components may be added, allowing for the inclusion of anisotropic dielectric properties, intuitive implementation of various electrode features, circuit representations of neurons in this bulk tissue model, or other phenomena that can be approximated as an additional circuit, such as the electrode-electrolyte interface [33] (Fig. S1).

### C. Tissue and electrode capacitance

Due to the low-frequency nature of stimuli (i.e. 95% of Fourier spectrum under 1 kHz, long pulse-width, and low pulse-frequency) in the simulations performed, introducing tissue capacitance to the model should yield negligible differences in results [15,18,20,26,34–40]. To validate this assumption, various models were simulated with capacitive tissue properties following Gabriel et al. Further, the basic validity of this assumption was analyzed by calculating the *τ*_RC_ of a Cole-Cole representation of tissue bio-impedance [41]. *τ*_RC_ is the time which a capacitor charges approximately 63% across a resistor and can be found by multiplying the resistance and capacitance in an RC circuit, or resistivity and permittivity in a volumetric model. Based on this analysis and simulation results presented later, it was deemed appropriate to take advantage of the relative computational simplicity of purely resistive models, and capacitive properties of bulk tissue were omitted.

A capacitive layer was added between the tissue and electrode, with a double-layer capacitance of 10 μF/cm^2^. The method for applying a specific capacitance across the interface at the electrode surface was to distribute the capacitance into parallel capacitors, with one applied to each node on the surface of the electrode. Each new capacitor was placed perpendicularly to the electrode surface and in parallel with tissue-electrode interface resistors. This was done without an additional spatial component, as the thickness of the electrode-electrolyte layer is much smaller than can be realized in our model with its current spatial resolution.

### D. Heterogeneous resistivity

**Fig. 1.**
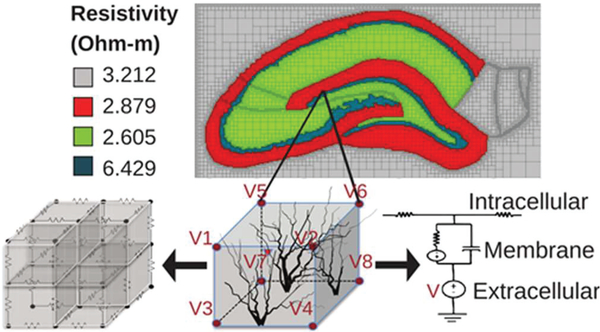
A conceptual depiction of the Admittance MethodNEURON model. A compartmental model of a neuron is represented as a cable model with compartments connected in series or in parallel according to the morphology. Each compartment is represented by a circuit model. Voltages calculated by the Admittance Method are applied as extracellular batteries to the appropriate compartments to stimulate a NEURON model. Experimentally determined resistivity values are used for subregions of the hippocampus [42].

**Fig. 2.**
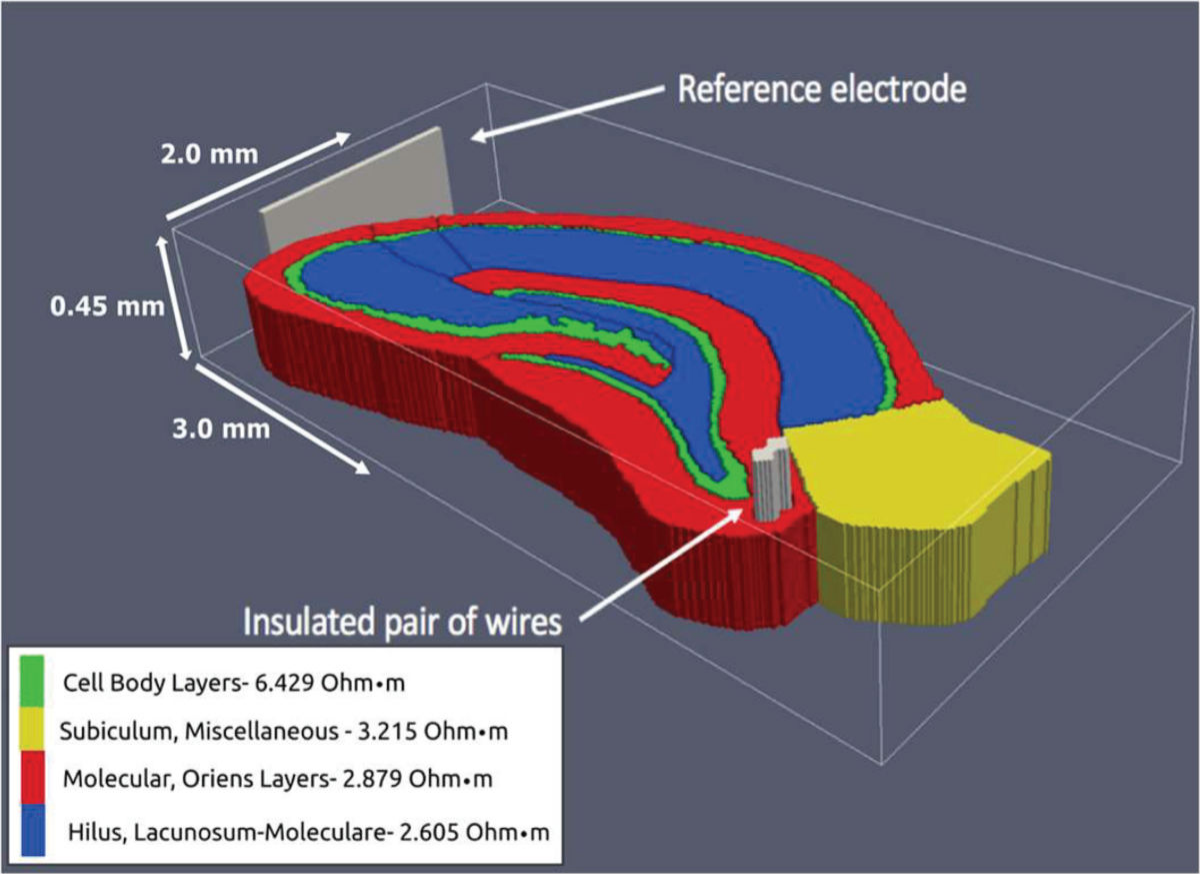
The resistivity values used for each section within the hippocampus [51].

The voxelized model was discretized based on the resistive properties of the hippocampal tissue at low-frequency (<10 kHz). Due to the unavailability of impedance measurements in the dentate gyrus, model resistivities were assigned based on measurements taken in CA1, using the four-electrode impedance measurement setup, by Lopez-Aguado, Ibarz, and Herreras [42]. Many measurements were performed by the experimenters in the apical and basal dendritic regions, cell body layer, stratum lacunosum-moleculare, and the hippocampal fissure bordering the outer boundary of the dentate gyrus. The measurements showed remarkably similar resistivity in the molecular regions, around 2.7 Ω m, with approximately double the resistance in the cell-body regions. This observation led to the simplification of the computational model, splitting the hippocampus into four regions, three molecular and one cell body, with each respective region having homogeneous resistive properties based on these measurements. The values implemented in the model are those reported by Lopez-Aguado et al. and are denoted in Figs. 1,2. Lopez-Aguado concluded that in the CA1 it should not be expected that a somatic to dendritic resistivity ratio be less than 2.22, though there is no clear indication whether a similar ratio can be expected in the dentate gyrus.

Because changes in this ratio would cause an increase in the gradient of the potential and may impact the activation of nearby cells, several simulations were performed with varying levels of heterogeneity. The original ratio of the cell body layer to molecular layer was 2.28 (control). This was varied in a series of simulations from cell body layer/molecular layer ratio of 1:1 to cell body layer/molecular layer ratio of 5:1.

To simulate electrical stimulation, two insulated microwire electrodes and a reference electrode were added to the model in replication of the work of Soussou et al. as shown in Figs. 1,2 [43]. The electrodes had a diameter of 48 μm and were given resistivity of platinum (10^−8^ Ω m). The insulation encasing the electrodes was 24 μm thick, with dielectric properties of teflon (10^16^ Ω m), and was cut flush with the surface of the electrodes. A bipolar current source was then applied, with the anode being placed at a node in the center of one of the micro-wires and the cathode in the other. All simulations were run with a bi-phasic, square-wave pulse, with a width of 1 ms. The results were tri-linearly interpolated and applied to each section in the large-scale compartmental (NEURON) model to predict induced neural activity. Potentials charge the capacitive membrane, resulting in intracellular currents.

### E. Overview of the large-scale NEURON model

The neural response to extracellular electric fields is simulated using a biologically realistic, computational model of a 400 μm coronal region of a rat dentate gyrus (DG). Assuming a total hippocampal length of 10 mm and 1,200,000 total GCs, the model comprises 50,000 granule cells (GC) and 10,000 entorhinal cortical (EC) axons, many features of which have been previously described [29,44]. The NEURON simulation environment (7.3) is used to simulate the compartmental models. The model is parallelized and run on a 4,040-processor computing cluster supported by the High-Performance Computing and Communications Center at University of Southern California.

### F. Granule cells

The morphologies of the GC models were generated using the L-NEURON tool [45]. Distributions of structural parameters were extracted from a database containing morphological reconstructions of GCs, and the distributions of parameters were used to create unique structures for the GC models [46]. For each generated morphology compartment lengths were kept uniform. The current morphologies include the dendrites and somas of the GCs but not the axons.

The biophysical parameters used in the GCs were derived from previously published computational models of the dentate gyrus [47,48]. These specifications include the types, densities, and distributions of the ion channels throughout the neuron model. The electrophysiological parameters and characteristics of the GC models used in this work have been previously validated [29].

### G. Entorhinal projections of the perforant path

While the eventual goal of this work is to model perforant path fibers along with their actively signaling parent cells, this current iteration models the perforant path as singly-branched cables with *in vivo* Hodgkin-Huxley biophysics. The most recent morphometric description of axons in the molecular layer of the DG report very narrow axons in spiny stellate EC cells, the most numerous cell type of the perforant path, with diameters as small as 0.1μm in between “periodic varicosities”, or thickenings, that likely correspond to presynaptic boutons [44]. The remaining topological and morphological features of the axons implemented in this model are simplistic approximations of the axon arbor statistics previously discussed [29]. A few other well-known and prominent features of the axons are implemented, namely an *en passant* connective schema, a primary bifurcation just above the crest of the DG, and the segregation of the medial and lateral perforant path, respectively, into the middle and outer thirds of the molecular layer of the DG. Proximity rules of connectivity between the perforant path fibers and GC dendrites are implemented to ensure appropriate conduction delays.

### H. Synapses

EC axons are connected to the GCs via 6,000,000 synapses in the outer third of the molecular layer for lateral perforant path axons, and 5,625,000 synapses in the middle third of the molecular layer for medial perforant path axons. Upon being triggered by an action potential, the synapse undergoes a change in conductance which allows current to flow into the post-synaptic compartment. The time course of the conductance (g) is approximated as the difference of two exponentials (Eq. 1):

(1) $$g=e^{\frac{-t}{\tau_{1}}}-e^{\frac{-t}{\tau_{2}}}$$

The postsynaptic response between entorhinal axons and GCs in this model is mediated exclusively by AMPA receptors. The maximal conductance for the synapse was chosen so that the amplitude of a single EPSP at the soma is 0.22 mV, with average *τ*_1,2_ of 1.05 and 5.75, respectively [48].

### I. Interface between NEURON model and Admittance model

Because the AM and NEURON models share the same coordinate space, the voltages from the AM are superimposed on the NEURON model to drive the compartments of the neurons. Through the addition of voltage sources in series with each membrane, extracellular potentials may be applied to neuronal compartments within the NEURON model using the following relationship (Eq. 2):

(2) $$I_{m}(t)=\frac{dV_{ext}}{dt}*C_{m}$$

Where *I*_*m*_ is the transmembrane current which results from an extracellular potential (*v*_*ext*_) charging the membrane capacitance (*C*_*m*_) Potentials were applied throughout GC models at the center of each NEURON section (20–30 locations), and each perforant path axon had voltage applied to 100 different locations evenly distributed throughout the extent of their structure. The circuit diagram for the application of the external voltage is shown in Fig. 1.

### J. Model Validation through LFP Prediction

**Fig. 3.**
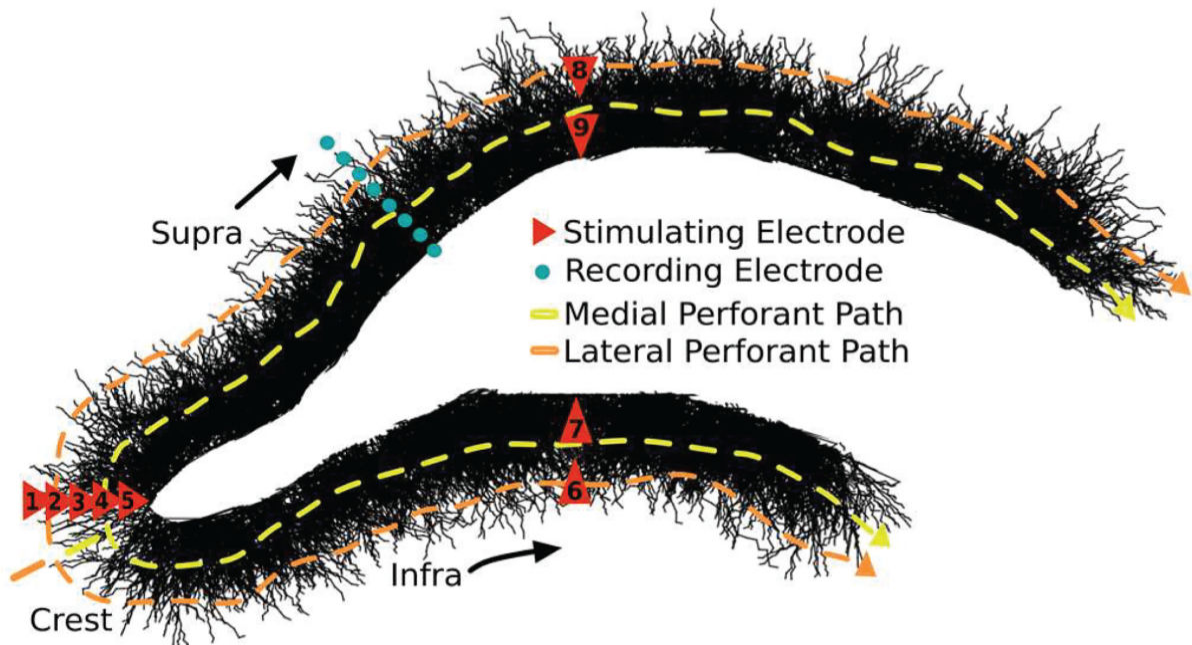
2-D rendering of the volumetric detailed neuronal model with demarcation of recording and stimulating devices. Notice the laminar, *en passant* topographical features of the perforant path; these are a strong determinant of the behavior of the tissue in response to extracellular electrical stimulation.

To evaluate the biological realism of the model, evoked potentials were estimated and compared with experimental recordings [43]. Transmembrane current densities were output at each simulated time-step of stimulation applied near the crest of the model DG (location 2 in Fig. 3). Stimuli used for comparison included biphasic, charge-balanced, and squarewave impulses of 1ms width and 200 A amplitude. Field potentials were then estimated for virtual point-electrodes located along the green dots in Fig. 3 using a variant of the line-source method, where resistivity was calculated as the path-length weighted average of domain resistivities (Eq. 3 and 4) [49,50].

(3) $$\phi (x,y,z)=\frac{1}{4\pi }\sum_{i=0}^{n}\frac{I_{i}}{K_{i}}$$

(4) $$K=\sum_{j=0}^{n}\sigma_{j}*r_{j}$$

Where Φ is the field potential resulting from a current source, I. K is the discrete integral of the product of tissue conductance and radial distance (sigma and r, respectively), where r is found by Eq. 5:

(5) $$r=\sqrt{{\left(x-x_{0}\right)}^{2}+{\left(y-y_{0}\right)}^{2}+{\left(z-z_{0}\right)}^{2}}$$

### K. Minimizing power required to achieve population spiking

A survey of stimulation parameters was conducted to determine which conditions best minimized power while still eliciting a concert of activity from the tissue. For each of the nine electrode locations, designated in Fig. 3, biphasic squarewave impulses (1 ms pulse width) were delivered over a range of amplitudes (50–650 μA) in increments of 50 μA. The network responses were analyzed for several features intending to thoroughly describe magnitude, time-course, and velocity of the population response. For this particular portion of the analysis direct activity, or spiking activity observed prior to the arrival of synaptic events nearly concurrently with the stimulus, was deliberately excluded. The remaining indirect, or synaptically driven, activity was measured for total proportion of cells active, maximum instantaneous proportion activated (2 ms time-bins), maximum slope of the network activity cumulative density function, as well as the half-height width of the indirect response.

**Fig. 4.**
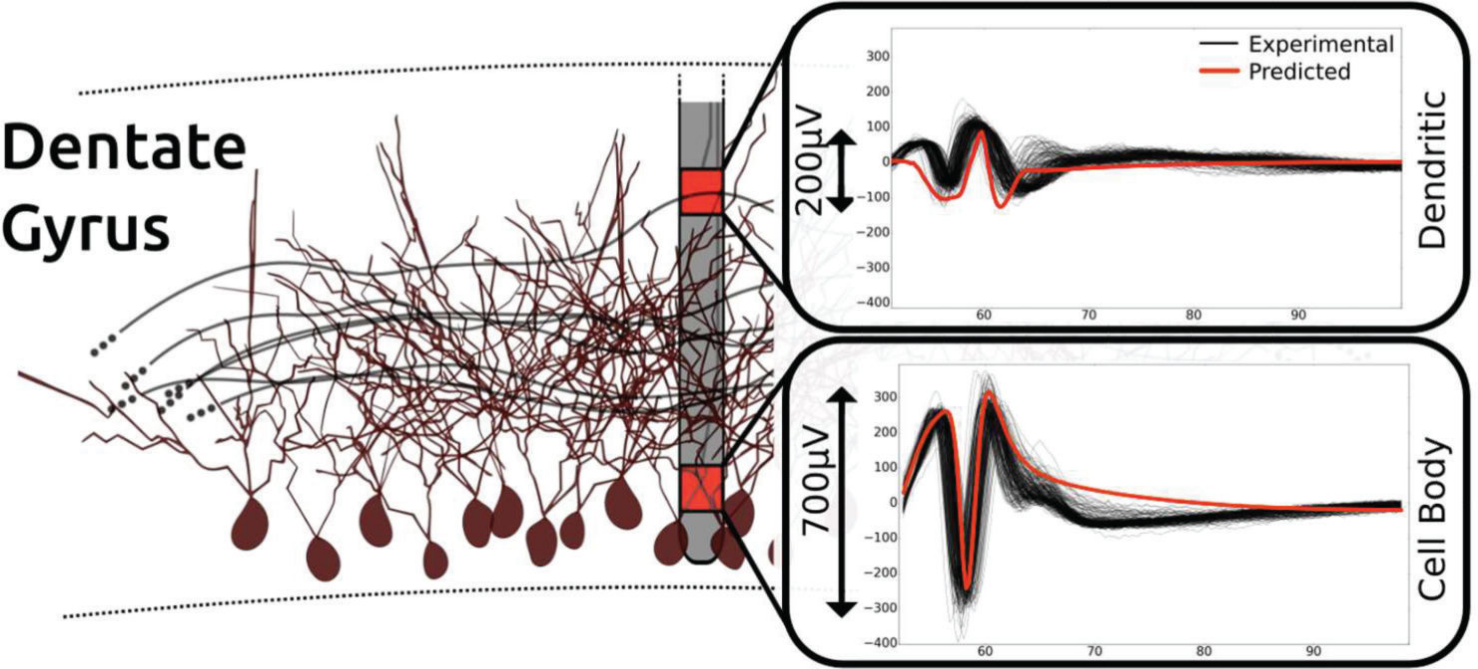
Predicted LFPs (magenta signal) exhibit a characteristic PS as observed in experimental evoked potentials (black signals) elicited by a 200 μA biphasic, square-wave pulse [43] . “Positive-going” spikes in the dendritic domain and “negative- going” spikes in the cell body layer indicate the emergence of a dipole. This is due to the laminar spatial separation of excitatory post-synaptic potential and action potential sites of potential and action potential sites of origination.

The concept of a population spike (PS) has been a popular one with electro-physiologists and especially those who work with the hippocampus. The architecture of the DG, characterized by the common orientation and dense packing of GCs, leads to a summing effect in the extracellular potentials recorded when EC afferents are stimulated concurrently [51]. Predictions made in the present study provide clues to the underlying electrical activity which results in PS observed in local field potentials. While this phenomenon can be observed via predicted evoked potentials (as in Fig. 4), having access to the membrane potential of each individual compartment in the NEURON model provides a level of detail only partially captured by experimentally recorded field potentials. For the purposes of these results, the PS is a synaptically activated and temporally phasic response expressed as an aggregation of spiking events observed in the model and not due to direct activation of granule cells in the dentate gyrus by the stimulation event. These two phases of the response are easily separable because of the delay associated with the axonal activation, synaptic conductance, and later suprathreshold activation of granule cells in the tissue. Fig. 6 demonstrates the features of the PS that are measured in this study, which combines 117 simulations over a range of stimulation amplitudes in 9 different electrode locations.

### L. Multi-objective evaluation of stimulation conditions

There are many ways a neural response could be characterized, but knowing which metric correlates with positive clinical outcomes or a successful experiment is impossible without defining the specific problem. The following metrics represent several important features that could generally be useful to optimize: a large PS amplitude would be necessary to ensure the successful transmission of the response to afferent areas; a short half-height width would allow greater temporal precision and potentially increase the temporal resolution at which a particular neural response could be encoded; finally, for a given pulse-width, a greater power efficiency could be important for safety and to increase the battery life of an implant. A simple, multi-objective optimization function was constructed using the weighted sums method to determine how each stimulation case would satisfy these constraints (Eq. 6).

(6) $$U=\frac{w_{1}PS_{max}+w_{2}PS_{efficiency}+w_{3}[max(HHW)-HHW]}{w_{1}+w_{2}+w_{3}}$$

Where *PS*_*max*_ is the peak proportion of GCs active, *PS*_*efficiency*_ is the number of GC spikes per unit stimulus, HHW is the half-height width of a probability density function fit to the instantaneous activity (Gaussian), and w1,2,3 are weights associated with each metric. Each metric is an independently normalized input to the multi-objective function. In this no-preference formulation, the weights were naively assumed to have a value of one, such that the average of the normalized metrics was computed. However, if the importance of each objective were defined by a decision maker or an automated method based on the situation, weights could be assigned a priori to yield different results. As formulated, high values of U indicate elicitation of a distinct and efficient population response (high peak activity, highly concurrent activity, and high activity per unit stimulus amplitude).

## III. RESULTS

Once the model was constructed, simulations were performed to determine general features of the response. The results of more specific studies performed after these test simulations comprise three main parts: (i) prediction of evoked field potentials, (ii) an analysis of model sensitivity to changing bulk tissue dielectric properties, and (iii) an improved stimulating parameter search where the AMNEURON model was tested for sensitivity to stimulating location and pulse amplitude with the goal of eliciting a power efficient PS.

### A. General spatiotemporal properties of threshold response to changing stimulating location

**Fig. 5.**
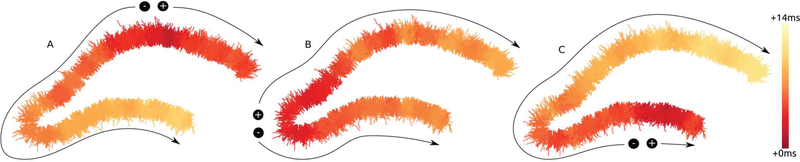
Heatmaps of spatiotemporal patterns of PS activity given the different locations of stimulation along the transverse axis (time calibrated from time of stimulation). (**A**, suprapyramidal PP at 100μA; **B**, crest PP at 150μA; **C**, infrapyramidal PP at 150μA). Activity along the transverse axis is strongly dependent upon the principle *en passant* topology of the perforant path.

**Fig. 6.**
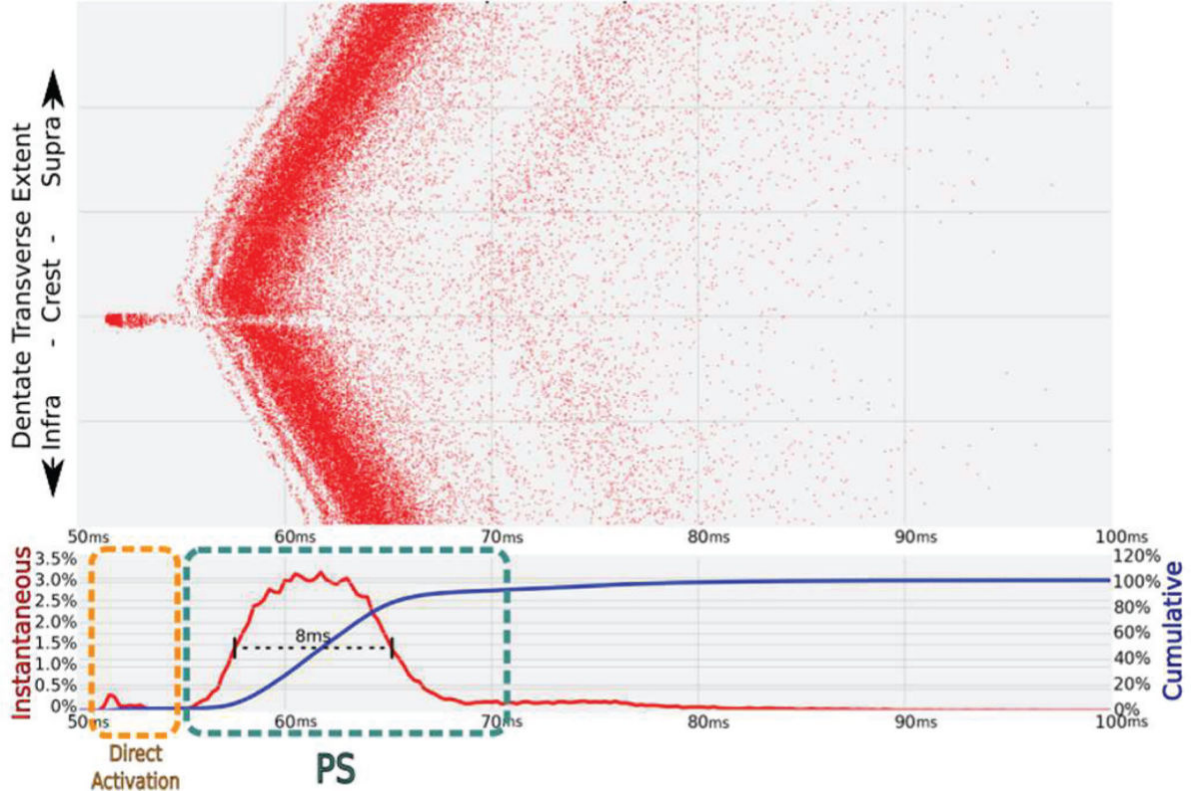
50k cell/10k axon network response to a 300 μA biphasic pulse (−/+) delivered by electrode two at time 50 ms. Each red dot represents an action potential at some location along the transverse axis of the tissue (large Y-axis) in time. Activity is also measured in terms of instantaneous (red line, 0.25ms bins) and cumulative response (blue). The total response is temporally separated into a direct activation phase (orange box) and a population spike (PS) phase (green box).

Initial simulations yielded some nearly universal commonalities: once a critical mass of PP axons reach threshold (always occurring in compartments most proximal to the leading cathodal source in this model) PS activity propagates in both directions along the transverse arc (Fig. 5,6). For Fig. 5, direct activation was excluded to emphasize the propagating PS. The chief difference remaining between perforant path and cell body stimulating locations is the activation threshold triggering the pattern that can be seen in these plots. For PP locations, PS propagates successfully along the transverse axis between amplitudes 100–150 μA, where cell body electrodes required amplitudes >300 μA. Importantly, the topography of PP-dentate connections leads to much greater concurrency and earlier termination of crest initiated PS.

### B. LFP Predictions and Qualitative Comparison with Experimental Recordings for Model Validation

**Fig. 7.**
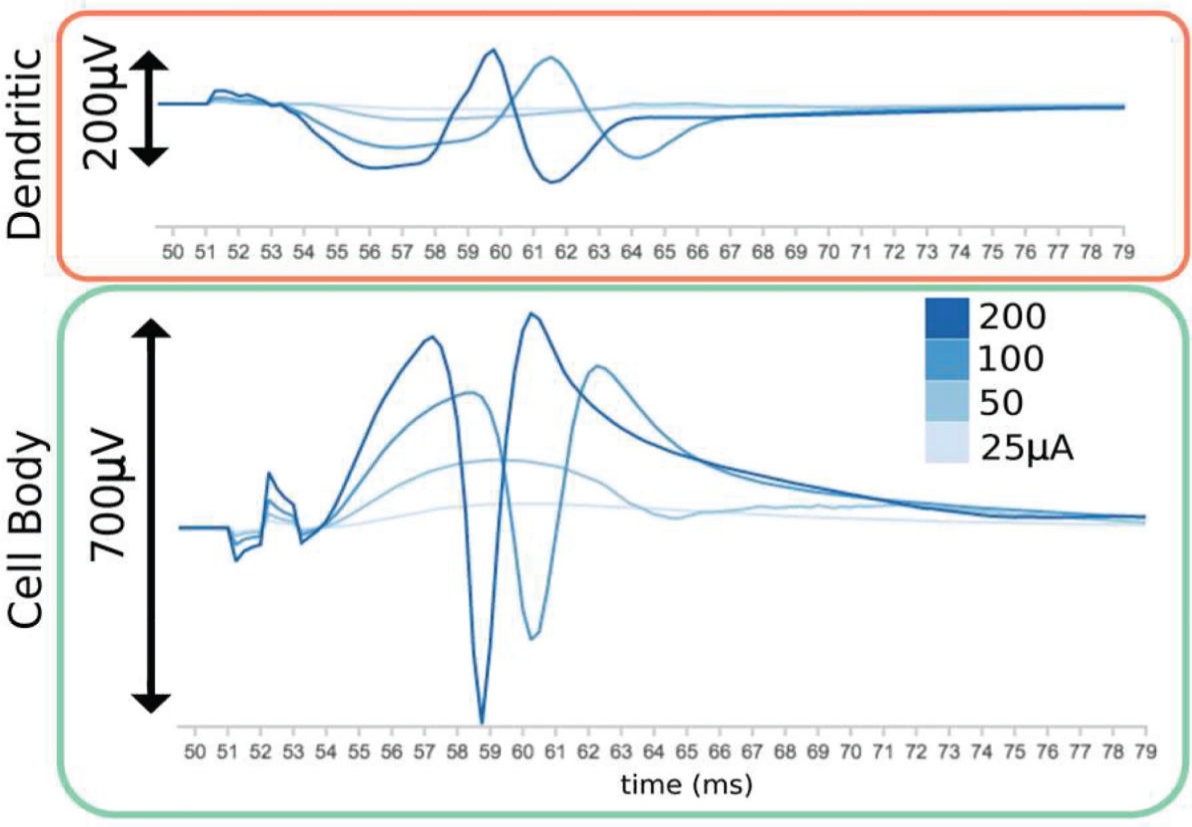
Predicted LFPs develop a dipole as stimulation amplitude is increased [43].

Predicted evoked potentials exhibit characteristic “negative-going” signals in the cell body layer and “positive-going” signals in the molecular domain (Figs. 4, 7). Upon comparison with recordings from experimental studies, predicted waveforms appear to capture the dipole behavior, PS amplitude, and time-course expected from the densely-packed and highly laminar granule cells [43, 51]. The development of a characteristic dentate evoked potential, with a dipole along the cell body-dendritic arbor axis, occurs gradually as stimulus amplitude is increased to 200 μA (Fig. 7).

### C. Sensitivity to changes in resistivity and capacitance

**Fig. 8:**
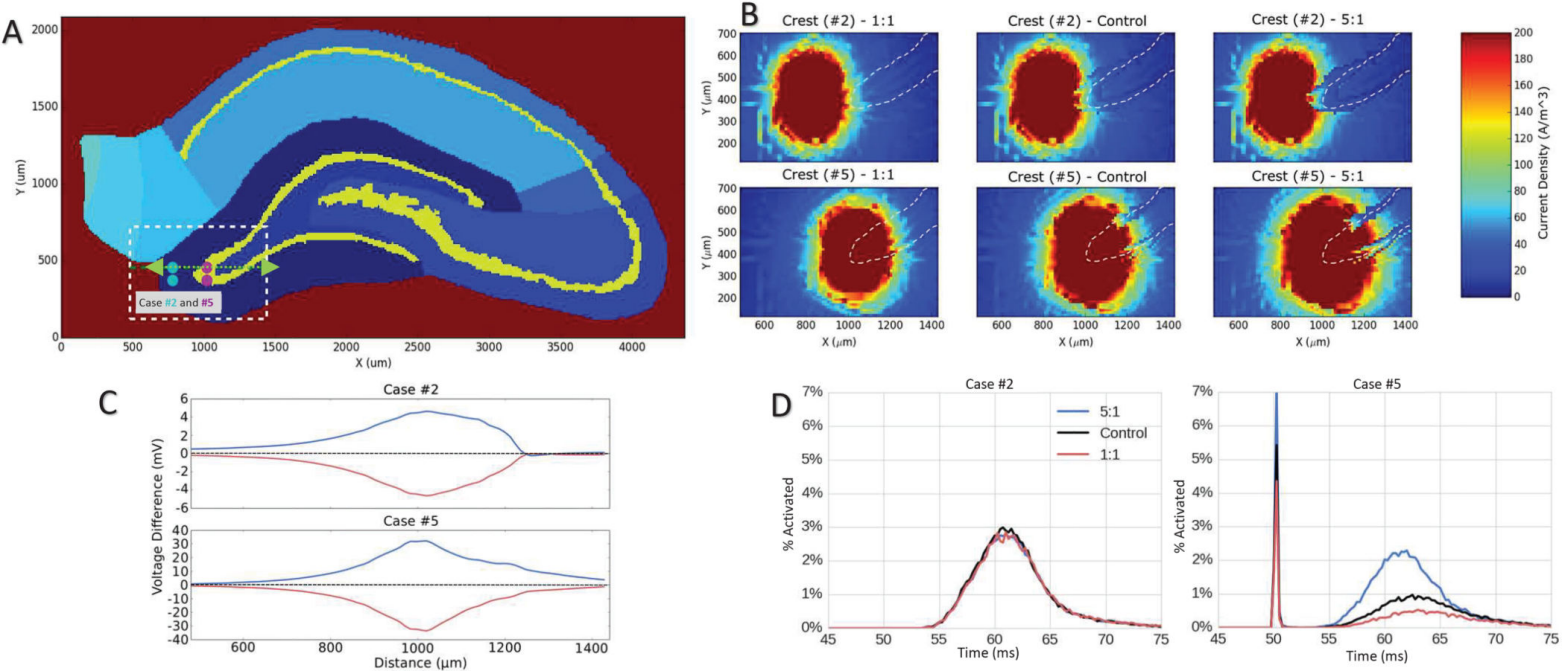
**(A).** The cell body layers are highlighted in yellow. The white bounding box shows the borders of the plots in Fig. 8.B. The greendashed-arrow segment is the base of Fig. 8.C. Cyan and magenta dots show the electrode locations for dielectric sensitivity analysis in theperforant path (case #2) and the cell body layer (case #5), respectively **(B).** Current density map for various levels of heterogeneity in a slice 18 μm beneath the electrode. Increasing the heterogeneity blocks the current from spreading into the cell body layer and the hilus toward the CA3 region. (Control = 2.28:1). **(C).** Shows voltage difference between each ratio of cell body to molecular layer resistivity experiment (1:1 in red, 5:1 in blue, control in dotted black ~2.28:1) and the control from left to right across the green dotted arrow in Fig. 8.A. Stimulating in the perforant path (top) results in smaller differences than does stimulating in the cell body layer (bottom). **(D).** Shows the NEURON model response to varying resistivity under otherwise controlled stimulating conditions for two electrodes placed at the crest. (case #2, left When stimulating in the perforant path of the crest, changes in cell body layer resistivity have a very limited impact on the features of PS. (case #5, right) When stimulating in the cell body layer of the crest, changes in resistivity can be seen to have a very large impact not only on PS features but also on the number of directly activated cells. (Control = 2.28:1).

Fig. 8.A can be referenced as an anatomical guide to results of the sensitivity analysis presented in 8.B, 8.C, and 8.D. Case #2 and Case #5 correspond to perforant path and cell body layer electrodes, respectively. Changes in the relative heterogeneity of the resistive properties of each region of the tissue being modeled results in measurable changes in the time-course, spatial distribution, and magnitude of the resulting response. Fig. 8.B demonstrates the difference in the spread of current as tissue heterogeneity is varied between 1:1 and 5:1 (control=2.28:1). In the homogeneous experiments, the current spreads uniformly through the tissue, penetrating well into the hilar region beyond the cell body layer. As the heterogeneity of the tissue is increased, the cell body layer inhibits current flow, acting as a conductive barrier, changing the gradient of the spread and lowering the density of current within the hilus. 7he normalized root mean squared error (NRMSE) of the voltage taken within the dotted bounding box shown in Fig. 8.A between each experimental case and the control was taken at three different depths within the slice. For the electrode placed in the perforant path, the NRMSE was less than 0.05 for all depths and was approximately the same for both the 1:1 and 5:1 experiments. With the electrode placed in the cell body layer the NRMSE increased to 0.17 for the 5:1 experiment and to 0.37 for 1:1. The NRMSE increases for the 1:1 case due to the increased spread of the current channeled through the cell body layer. The changing dielectric properties primarily result in changes of potential in the cell body layer but changes also occur in the hilar region (Fig. 8.A in conjunction with Fig. 8.B).

Fig. 8.C shows how the magnitude of the difference between each experiment and the control test varies across the magenta axis in Fig. 8.A. Placing the stimulating electrode within the molecular layer of the crest led to a maximum difference of approximately 10 mV within the cell body layer for both the cell body layer/molecular layer 1:1 ratio and cell body layer/molecular layer 5:1 ratio experiments. However, placing the electrodes within the cell body layer led to an increase to 60 mV difference. The maximum difference in voltage for the latter experiment was several times larger, indicating that increased heterogeneity could lead to a larger difference in model results for this electrode placement.

When these cases were simulated in the NEURON model, the differences were more apparent (Fig. 8.D). When considering the stimulation of the cell body layer of the crest, the homogeneous case resulted in a net decrease of activity in all regions of the NEURON model while increases in the resistivity of the conductive barrier feature may ultimately result in increasingly distal sites of direct activation at a given stimulation amplitude. The 5:1 resistivity ratio case yielded a net increase of total activity.

Regarding the inclusion of capacitance in the AM field estimation step, for the model parameters outlined in section the II.C, differences in the field potentials of less than 0.1% throughout the modeled tissue were observed in comparison to the model with no tissue capacitance when each model was stimulated with a biphasic current pulse of 100 μA magnitude and 1 ms in duration. Further, this assumption is intuitive when considering the *τ*_RC_ of a Cole-Cole representation of tissue bioimpedance [41]. The *τ*_RC_ provides a simplified estimate of what stimulating currents might be suitably approximated by purely resistive models. Where *τ*_RC_ is much less than the pulse-width of a current-controlled stimulus, it can be assumed that tissue capacitance is a negligible determinant of the potentials around the circuit. For a 1 ms current impulse, with a resistivity of 2.5 Ω m and permittivity of 2.79 × 10^−6^ F/m, capacitors in the volume would charge to 95% in under 21 μs. A complete record of these experiments can be found in supplemental materials (Fig. S2).

### D. Minimizing power required to achieve population spiking

**Fig. 9.**
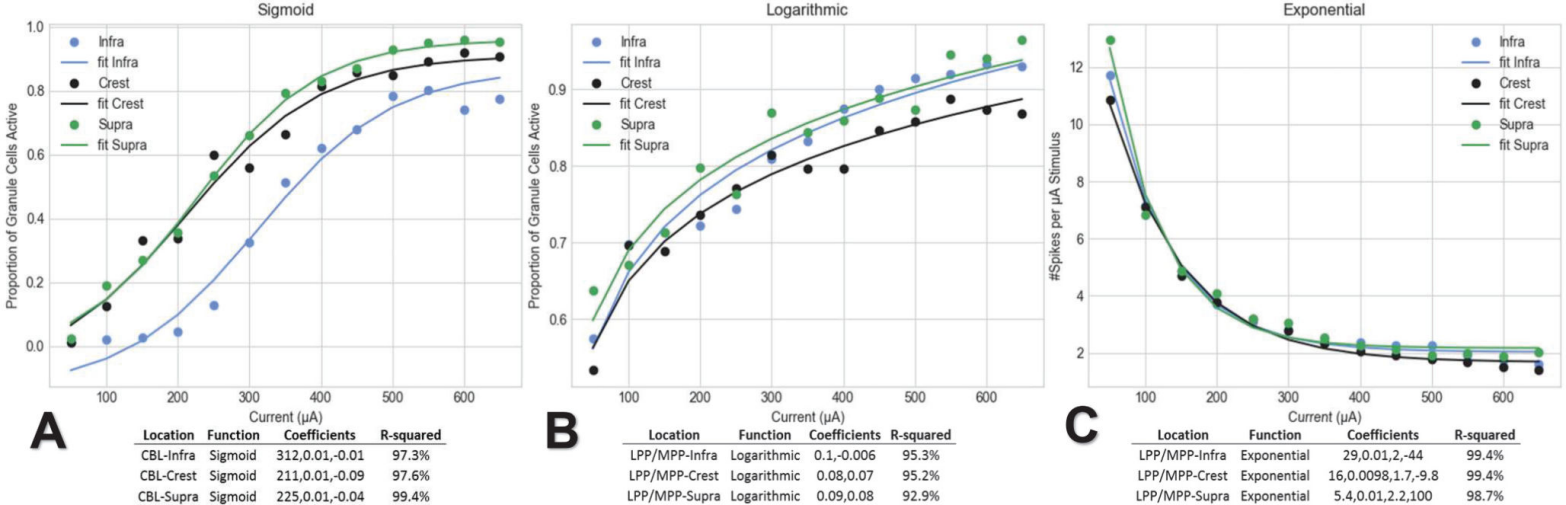
Spiking activity at most locations saturates above stimulus amplitudes of ~500 A. (A) When stimulating at cell body locations, granule cells activity versus stimulus amplitude follows a sigmoidal trend. (B) When stimulating at PP locations, the trend is more logarithmic. (C) The differences between (A) and (B) gives rise to greater efficiency at low amplitude when stimulating in the PP.

**Fig. 10.**
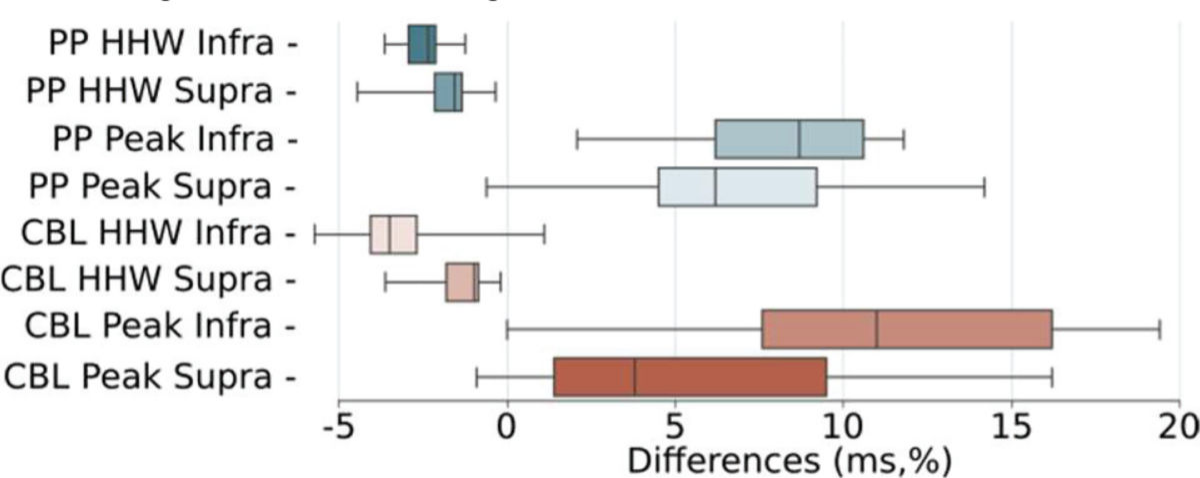
For nearly all stimulating conditions the half-heightwidth (HHW) of PS was shorter (ms) when stimulating at the cellbody layer and PP at the crest relative to the supra and infrapyramidal transverse locations. While more variable, nearly all stimulating amplitudes resulted in larger peak PS amplitudes (%) at the crest relative to supra/infra locations

**Fig. 11.**
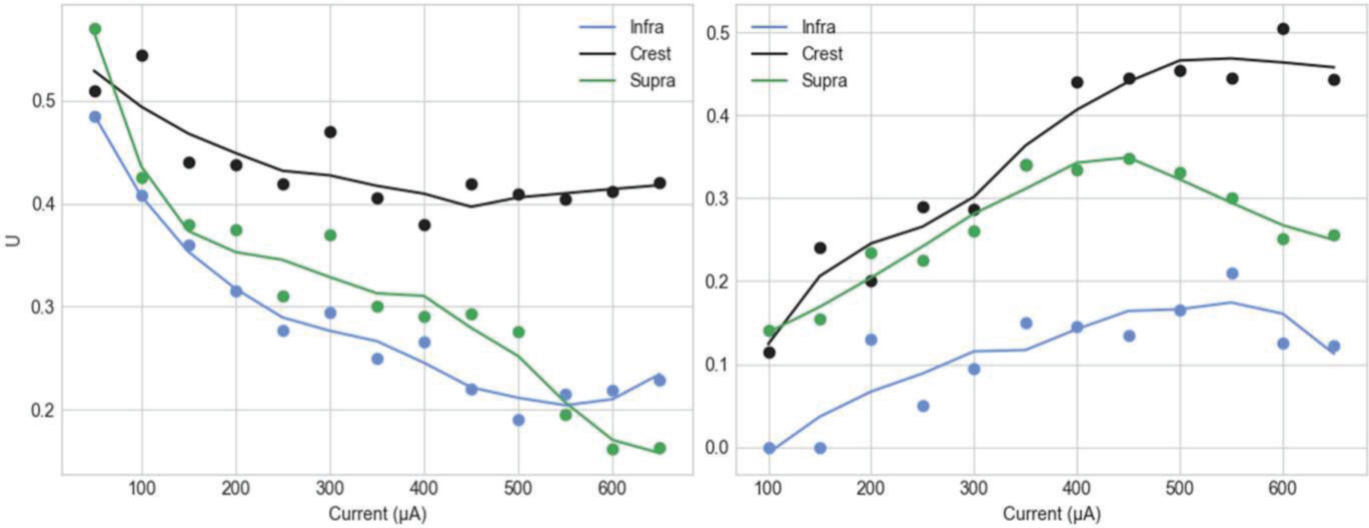
Output of the multi-objective optimization function for MPP/LPP (left) and CBL (right) stimulation cases. High values of U indicate strong PS. The population spike amplitude and power efficiency were maximized and the half-height width was minimized with equal weighting in this optimization. (CBL-cell body layer, LPP/MPPlateral/ medial perforant path). Trend-lines, added to aid interpretation, are created using a 3^rd^ order Savitzky-Golay filter.

A key goal of this modeling effort was to discover improved electrode and stimulation protocols for hippocampal tissue. This study presents results of a thorough bilinear optimal parameter search, where an improved electrode location and stimulating amplitude is selected with which to elicit a power efficient PS. The critical results of these experiments are presented in Figs. 9, 10, and 11. A complete record of these experiments can be found in supplemental materials (Fig. S3-4).

### E. Recruitment of axons and GCs

The response of the tissue to increasing stimulation amplitude for any location was nonlinear. While it seemed that axonal recruitment increases in a disjointed manner, there was a sigmoidal shape to response curves when stimulating in the cell body layer (9.A), where PP location recruitment pattern was more obviously logarithmic with increasing stimulus amplitude (9.B). The difference arises from lower thresholds for GC recruitment in PP locations. Despite unremarkable axonal activation, it was notable that a slightly larger proportion of GC activity was seen in suprapyramidal blade stimulating locations.

### F. Population spike peak amplitude and sharpness

The population spike peak amplitude and half-height width were calculated by counting the number of spikes generated by the GCs within 2 ms bins. The peak amplitude was normalized by the total number of GCs and converted into a percentage. The half-height width corresponded to the total time during which the population activity was above half of its peak amplitude. The proportion of granule cells active at peak PS was highly location dependent, with a markedly greater number active at the crest. As stimulation amplitude increases there is a general increase in peak proportion, but there was some drop-off in several cases above 500 μA. This drop-off was exaggerated in the suprapyramidal stimulating locations where it occurs earlier and more aggressively.

Electrodes at the crest were notable for both larger amplitude and shorter half-height width PS from the tissue model (Fig. 10). There is no clear trend in PS half-height width (measured in ms) as stimulation amplitude increases across all locations but there are some electrodes, particularly at the crest and cell body layer in the suprapyramidal blade where there was a strongly sloping, and supra-linear shortening of PS half-height width.

### G. Power efficiency of activity

Power efficiency was calculated by normalizing the peak PS amplitude and the total proportion of GCs activated by the stimulation amplitude. For locations along the transverse axis, as the stimulating current source is increasingly centered in between the lateral and medial perforant path the response per unit stimulus exhibits an exponential trend (9.C). Stimulating at the cell body layer, however, finds a maximum efficiency at midrange amplitudes rather than high or low. The greatest efficiencies are seen in perforant path locations below amplitudes of 250 μA, without regard for transverse extent. These data were then used as inputs to Eq. 6 to calculate U for all stimulus location/amplitude pairs. Fig. 14 demonstrates the PS efficiency is best at MPP/LPP locations of the crest at low amplitudes (~50–100 A).

## IV. DISCUSSION

As a first step toward modeling the entire hippocampus, a model encompassing a section of the dentate gyrus that also includes the perforant path axons has been completed. Where other studies have focused on either the proper implementation of bulk-tissue-level modeling without network dynamics or the results of much smaller networks using multicompartmental neuron models, this study demonstrates how a hybrid, multi-scale AM-NEURON model reveals properties of hippocampal activity which only emerge with both scale and detail. Studying the sensitivity of models, like those described in this paper, to variations in dielectric properties and electrical stimuli will reveal fundamental characteristics of the hippocampal system. This knowledge guides design of more effective therapeutic interventions and devices.

### A. Improvements of the model compared to previously published work

By scaling the AM-NEURON method up to the tissue level, the model demonstrates the capability to predict DG response to a variety of configurations of stimulating electrodes and stimuli. Previous studies based on the NEURON model used in this work have reported the existence of naturally emerging spatiotemporal clusters of activity in the DG due to synaptic input [29]. This new model uses a digitized three-dimensional hippocampal atlas to preserve the overall anatomy of the macrostructure and the hippocampal subfields. This model adds the ability to provide an additional source of input, via electrical stimulation, and includes explicit axons for the EC projections. These additions grant the ability to explore the temporal and spatial components of localized PS by allowing a precise view of active propagation along the transverse extent of the structure, an unexplored topic due to the highly divergent nature of the excitatory EC inputs. The inclusion of actively conducting axons that follow an anatomically correct topography is an addition that has allowed more powerful prediction of the spatiotemporal response to extracellular stimulation; where simplistic activating functions might have only successfully predicted the immediate and local response of directly activated cells (i.e. axonal sites of action potential initiation) a hybrid, multi-scale networked AM-NEURON model can also show how patterns of activation spread due to the initial response to the stimulation *and* the subsequent synaptic events that are triggered by propagating action potentials. GC action potentials can be generated along the transverse axis in locations several hundred microns away, where stimulating potentials approach zero.

### B. Computational Resources and Considerations

Computational burden is an important challenge to consider when choosing the modeling approach outlined in this study. Successful simulation of large networks of detailed neuronal models requires considerable computational resources. To make this study possible, the model was parallelized to take advantage of a 4,040 processor HPC cluster owned by the authors. The simulations reported in Figs. 5 and 6 were run on 600 processors and each took 3.5hrs. to complete, producing 45 GB of data per stimulation case. The PS sensitivity analysis consisted of 117 simulations of a scaled down (1:25 GCs) version of the model presented in Figs. 9, 10, and 11 were run on 60 processors. These simulations produced approximately 200 GB of data and required nearly 585 hours of processor time to simulate just under 12 seconds of network activity. Despite the interpretability of parametric models, ever increasing model complexity poses significant computational challenges. Future models with more complete topology, longer simulations, complex stimulation protocols, and incorporation of feedback potentials, will likely require more resources than can be brought to bear without further optimization. Methods to reduce the computational burden include nonparametric multiple-input/multiple-output modeling, algorithms to simplify dendritic and axonal branching complexity, alternative numerical or analytical volume conductor methods, and GPU reimplementation [52].

### C. Bulk Tissue Capacitance and Current Division at Granule Cell Layer

Because of the unique anatomy of the hippocampus, it was clear that careful consideration of the dielectric properties of bulk neural tissue would be critical to performing accurate prediction of the network response.

While there is previous evidence of the negligible contribution of capacitance at low-frequencies (≈1 kHz in this study), this assertion was affirmed for more numerously populated neuronal models than has been published [26].

The ability of the AM to create a complex heterogeneous model is important in the hippocampal system because of how the granule cell layer alters the path of injected current, to avoid regions of high resistivity and changes the gradient of the electric field. Results from a resistivity ratio sensitivity analysis demonstrate the large impact that changes of this property can have on the network level activity of a complex tissue system. As the relative resistivity of the cell body layer was raised, the conductive barrier became more apparent. This study illuminated the role the DG cell body layer could play in shielding hilar structures and how dynamic changes in resistivity could make this region more or less vulnerable to stimulating current penetration. While this study presents some exploration of the impact of varying tissue resistivity and capacitance, the dynamic nature of neural tissue dielectric properties warrants further study [52,53].

### D. Power efficient elicitation of population spiking

In the 1970’s, Sam Deadwyler and others popularized the measurement of PS as a metric of hippocampal tissue response to electrical stimulation [51]. This observation in local field potentials was adapted as a rough approximation of the quantity and concurrency of EPSP in granule cells. The present model and the analysis performed was designed to provide a similar, though much higher resolution, quantitative evaluation of the activity elicited by electrical stimulation. The proxy measurements of PS used in this study demonstrate how this model could be used to determine an improved and power efficient device and impulse with which to elicit PS from the principal cells of the DG.

The clearest conclusion to be made from the series of 117 simulations that explored the sensitivity of the hybrid model to stimulation location and amplitude is that proximity to dense regions of axons is the strongest physical system determinant of PS threshold. As stimulating electrodes were moved closer to the zone between the lateral and medial perforant path, regardless of transverse extent, a dramatic decrease in activation threshold was seen (Fig. 9.A/B). In this region, the electrode distance to thoroughfare axons in the tissue system is minimized. Second only to axonal proximity in determining PS size was transverse location; electrodes placed at the crest saw both a marked increase in peak amplitude and a decrease in half-height width of PS (Fig. 10).

Also significant are the non-linear relationships between the system response and stimulus amplitude. The magnitude of GC response to increasing stimulation amplitude follows a roughly sigmoidal/logarithmic trend where high sloping regions occur at amplitudes where large recruitments of axons occur and low sloping regions occur where either few axons are near threshold or most axons are already suprathreshold (Fig.9.A/B). This indicates that there could be regions of diminishing returns where each unit of stimulus amplitude results in progressively smaller increases in the number of GC spikes. The decaying exponential trend seen in the perforant path response (Fig. 9.C) indicates that fewer and fewer spikes are yielded with each increase in amplitude across the whole range. This result indicates that power can be minimized for any arbitrary PS size or half-height width.

When combining these observations from the analysis (as achieved by Fig. 11) it is clear that if an experimentalist desired to elicit an efficient PS of respectable amplitude and minimal half-height width then such could be achieved by stimulating between the lamina of the lateral and medial perforant path at the crest of the DG at an amplitude as low as 100 μA, where this location is already achieving a clear and sharp PS, but not greater than approximately 250 μA when the efficiency drops off considerably.

Stimulating amplitudes in this paper are sometimes greater than those typically used in clinical applications, especially for very large and myelinated fibers. Activation thresholds could have been much greater that those presented given that very small unmyelinated axons are, theoretically, much less excitable [26,28]. It should be noted that this study adds to evidence that near-ideal conditions allow large evoked potentials to be elicited with 1ms pulses lesser than 300 A. However, not all stimulating locations studied in this paper had such low thresholds of excitation and these response curves did not begin to saturate until much higher amplitude pulses were used (i.e. cell body layer of the infrapyramidal blade).

### E. Model Limitations and Future Developments

First, this study presents predictions of evoked potentials using a different volume conductor method than that used to estimate the field resulting from stimulation. While each method is valid and appropriate in application, a more compelling implementation would be to use the same framework for both stimulus and response field estimation. In future work, the links between bulk tissue and neuronal hierarchical scales will be strengthened by allowing neuronal feedback potentials to flow between neighboring cells on a time-stepping basis. In addition to increasing methodological coherence, this feature would allow a thorough exploration of ephaptic coupling and other activity-dependent extracellular dynamics in various parts of the hippocampus [53,54].

As a preliminary work, the extent of the dentate system was limited to a 400μm section of tissue due to the complexity of the hybrid model. The end goal of the work will be to expand this section to encompass the entire 10–12mm extent of the dentate gyrus and incorporate other hippocampal layers. At this stage the effect of anatomic intricacies on the intracellular propagation of electric fields can be fully appreciated.

Finally, there are neural components present in the DG that are yet to be implemented in the model. Chief among these are dentate interneurons which would also be activated by electrical stimuli and interact with the granule cells to change the population dynamics. While feedback activity isn’t likely to occur fast enough to strongly impact the initial population response of resting tissue to a single 1 ms impulse in the perforant path, they may play a larger role in shaping responses to stimulation nearer the granule cell bodies where these cells synapse. Implementing these network components are a high-priority going forward so responses to sequences of stimuli can be studied.

## V. CONCLUSION

This study shows that the model performs the minimum functions necessary to improve electrical stimulation systems. However, future iterations of the model and methodology will be improved in several areas. While the list of limitations and proposed improvements is not exhaustive, incorporating these features will provide a powerful framework which can aid the design of devices capable of improved interfacing with this region of the brain. As the detailed neuronal componentry of the model is further expanded to include more cell types and layers of tissue it will provide a deeper understanding of both the network properties of the hippocampal circuit and better strategies of inducing and recording activity via arrays of electrodes. The implications of this work are far reaching as the community seeks better methods of developing and testing early iterations of prostheses for the treatment of diverse neurological disorders.

## Supplementary Material

1
