# Supplementary material for: Model-Based Analysis of Electrode Placement and Pulse Amplitude for Hippocampal Stimulation
Source: IEEE Trans Biomed Eng. Author manuscript; Available in PMC 2019 Oct 1. (PMC6224291; doi:10.1109/TBME.2018.2791860)
Supplement: 1 [file NIHMS1507698-supplement-1.pdf]

VI. Supplementary Materials

The following materials have been provided as a supplement to the primary manuscript. These materials include: (i) a detailed description of the approach to modeling the tissue-electrode interface, (ii) raster plots of the NEURON response to changing electrode depth and cell body layer resistivity near the crest of the dentate, (iii) complete tabulated results of stimulus location and pulse amplitude sensitivity analysis, and (iv) the full evaluation of these results via the multi-objective optimization function described in 2.L. These and core elements of the model have also been made available on ModelDB for reference. Should additional information prove essential to the understanding of a reader, please contact the corresponding author.

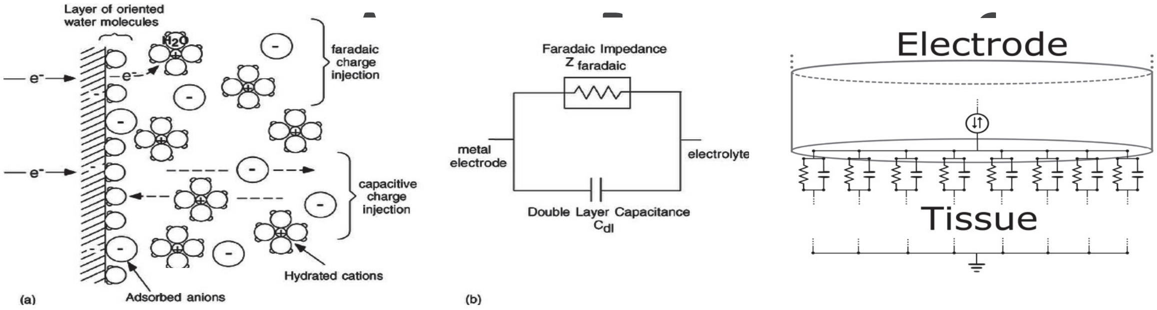

Fig. S1. The two modes of charge injection, faradaic and capacitive (A), are accurately modeled by parallel resistors and capacitors (B) (Merrill, D. R., Bikson, M., & Jefferys, J. G., 2005. *Journal of neuroscience methods).* Faradaic impedance and double layer capacitance at the tissue interface of physical electrodes with complex geometry can be spatially discretized (C).

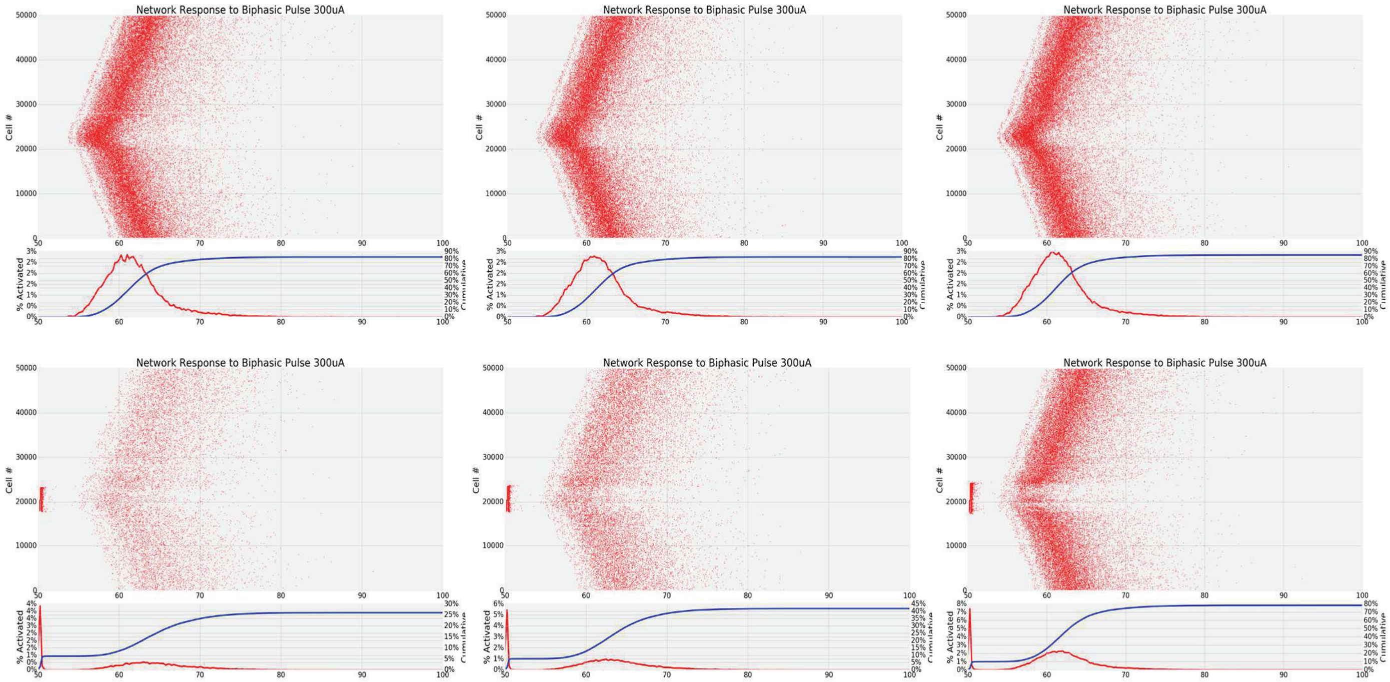

Fig. S2. Shows the NEURON model response to varying resistivity under otherwise controlled stimulating conditions for two electrodes placed at the crest. (case #2, top row) When stimulating in the perforant path of the crest, changes in cell body layer resistivity have a very limited impact on the features of PS. (case #5, bottom row) When stimulating in the cell body layer of the crest, changes in resistivity can be seen to have a very large impact not only on PS features but also on the number of directly activated cells. (Control = 2.28:1).

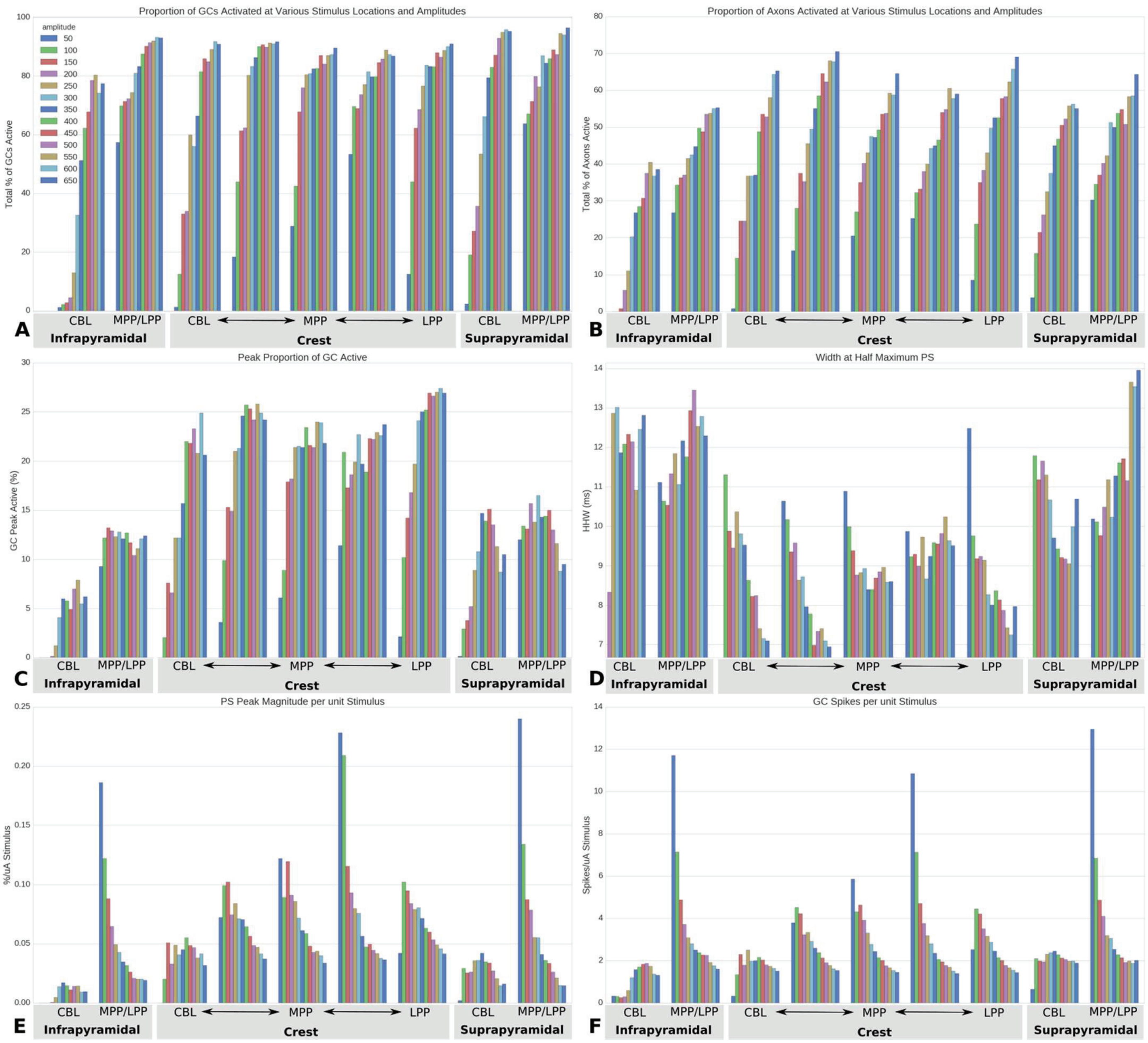

Fig. S3. 117 simulations of nine electrodes at amplitudes 50 µA to 650 µA in increments of 50 µA. (A) Nonlinear increases in GC activity correspond to axons recruitment and consequent synaptic activity. (B) Nonlinear recruitment of axons as stimulation amplitude increases is due to laminar topography. The medial/lateral performant path (MPP, LPP) of the infra-pyramidal blade and crest show a sigmoidal trend in GC response to increasing stimulation. (C) The proportion of GCs active at the peak of PS is location dependent with higher amplitudes at the crest due to greater concurrency of transverse propagation (D). Due to the greater concurrency of propagating activity, crest stimulation generates a shorter half-height width. (E) High concurrency of propagation along the transverse extent when stimulating at the crest results in greater amplitude PS per unit stimulus. Maxima indicate optimal stimulation amplitudes. (F) Electrode proximity to dense regions of axons results in activation threshold depression. Electrodes placed between the lateral and medial perforant path in the molecular layer of the tissue have greater activity for all amplitudes relative to the cell body layer (CBL).

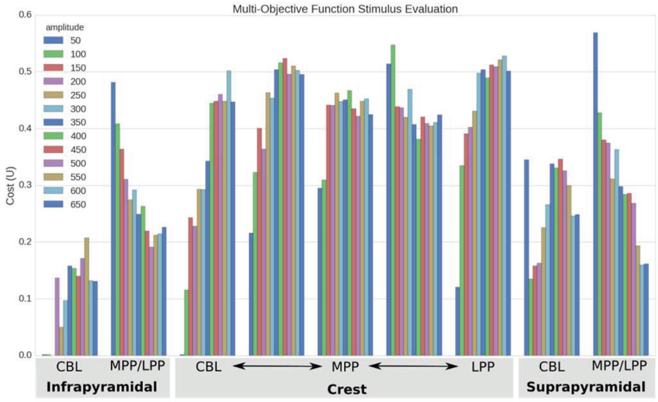

Fig. S4. Output of the multi-objective optimization function for all stimulation cases. The population spike amplitude and power efficiency were maximized and the half-height width was minimized with equal weighting in this optimization. (CBL-cell body layer, LPP/MPP-lateral/medial perforant path).
